# Inflammatory bowel disease increases the risk of hepatobiliary pancreatic cancer: A two‐sample Mendelian randomization analysis of European and East Asian populations

**DOI:** 10.1002/cam4.6057

**Published:** 2023-05-15

**Authors:** Jinsheng Huang, Xujia Li, Jicheng Hong, Lingli Huang, Qi Jiang, Shunqi Guo, Yuming Rong, Guifang Guo

**Affiliations:** ^1^ VIP Department Sun Yat‐sen University Cancer Center Guangzhou China; ^2^ State Key Laboratory of Oncology in South China Sun Yat‐sen University Cancer Center Guangzhou China; ^3^ Collaborative Innovation Center for Cancer Medicine Sun Yat‐sen University Cancer Center Guangzhou China; ^4^ Department of Emergency Shantou Central Hospital Shantou China

**Keywords:** Crohn's disease, hepatobiliary pancreatic cancer, inflammatory bowel disease, Mendelian randomization, ulcerative colitis

## Abstract

**Background:**

Both inflammatory bowel disease (IBD) and hepato‐pancreato‐biliary cancers (HPBC) have been established to cause a huge socioeconomic burden. Epidemiological studies have revealed a close association between IBD and HPBC.

**Methods:**

Herein, we utilized inverse‐variance weighting to conduct a two‐sample Mendelian randomization analysis. We sought to investigate the link between various subtypes of IBD and HPBC. To ensure the accuracy and consistency of our findings, we conducted heterogeneity tests, gene pleiotropy tests, and sensitivity analyses.

**Results:**

Compared to the general population, IBD patients in Europe exhibited a 1.22‐fold increased incidence of pancreatic cancer (PC) with a 95% confidence interval (CI) of 1.0022–1.4888 (*p* = 0.0475). We also found a 1.14‐fold increased incidence of PC in Crohn's disease (CD) patients with (95% CI: 1.0017–1.3073, *p* = 0.0472). In the East Asian population, the incidence of hepatocellular carcinoma (HCC) was 1.28‐fold higher (95% CI = 1.0709–1.5244, *p* = 0.0065) in IBD patients than in the general population. Additionally, ulcerative colitis (UC) patients displayed 1.12‐fold (95% CI: 1.1466–1.3334, *p* < 0.0001) and 1.31‐fold (95% CI: 1.0983–1.5641, *p* = 0.0027) increased incidences of HCC and cholangiocarcinoma (CCA), respectively. Finally, the incidence of PC was 1.19‐fold higher in CD patients than in the general population (95% CI = 1.0741–1.3132, *p* = 0.0008).

**Conclusion:**

Our study validated that IBD is a risk factor for HPBC. This causal relationship exhibited significant heterogeneity in different European and East Asian populations.

## 1 INTRODUCTION

Inflammatory bowel disease (IBD) is a recurrent inflammatory disorder involving the gastrointestinal tract^1^, ^2^ and can be classified into Crohn's disease (CD) and ulcerative colitis (UC).^3^ A previous epidemiological survey reported that IBD affects about 6.8 million people globally.^4^ Before the 21st century, IBD incidence was relatively high in the West (Northern and Western Europe and North America). However, increased incidence in the Asia‐Pacific region (including China) has been observed following industrialization and urbanization.^5^, ^6^, ^7^, ^8^ The etiology of IBD remains elusive and is widely thought to result from the interplay of several factors. There is an increasing consensus that IBD is closely related to genetic susceptibility, environmental factors, intestinal flora, intestinal mucosal function, immune response, oxidative stress, and inflammatory response.^2^, ^9^, ^10^, ^11^, ^12^ Hepato‐pancreato‐biliary cancers (HPBC), including hepatocellular carcinoma (HCC), cholangiocarcinoma (CCA), and pancreatic cancer (PC), are common malignant tumors of the digestive system. HCC is widely acknowledged as the predominant subtype of primary liver cancer, followed by intrahepatic cholangiocarcinoma and extrahepatic cholangiocarcinoma. Indeed, HPBC has long been established as the common malignant tumor in humans, with high malignancy and mortality rates.^13^, ^14^, ^15^

Overwhelming evidence from epidemiological studies substantiates a close association between IBD and HPBC. For instance, a case–control study by Yuan et al.^16^ found a heightened risk of PC development in UC populations (OR = 1.18, 95% CI: 1.07–1.31). Another study found a 30% and 10% higher risk of invasive cancer in CD and UC patients in the long‐term compared with the general population. Besides, the risk of CCA and HCC in IBD patients was significantly increased, and cancer risk increased during early disease.^17^ Taken together, our findings suggest a higher risk of HPBC in IBD patients than in the normal population. However, further research is warranted to determine their potential causal association.

Mendelian Randomization (MR) analyses involve using genetic instrumental variables (IVs) to draw causal interferences. Single nucleotide polymorphisms (SNPs) are usually used to infer causal relationships between exposure and outcomes due to their random at conception and are not subject to confounding, which minimizes confounding and reverse causality. Therefore, MR analysis provides stronger causal inference evidence than traditional observational studies.^18^, ^19^

To our knowledge, no MR analysis has hitherto investigated the potential presence of a causal association between IBD and HPBC. To that end, we conducted this MR study to establish whether there is a causal relationship between IBD and HPBC. Moreover, we sought to analyze the differences between Western (European) and East Asian populations. Importantly, our findings provide the foothold for more rational cancer surveillance programs focusing on patients with IBD, improving the timely identification of cancer and precancerous abnormalities, and reducing the health‐care burden.

## 2 MATERIALS AND METHODS

### 2.1 Data sources

The genome‐wide association studies (GWAS) data was obtained from the International Inflammatory Bowel Disease Genetics Consortium (IIBDGC), UK Biobank, PanScan1, and BioBank Japan. The IIBDGC is a global network of hundreds of researchers from 20 countries on four continents working on the genetics of IBD. The UK Biobank is an open‐access genetic and health information database on approximately half a million participants (aged 40 to 69) from the United Kingdom. The PanScan1 consortium utilized 12 prospective cohorts to perform GWAS and analyze the pooled PC data on 1896 cases and 1939 controls. Meanwhile, BioBank Japan compiled DNA, serum and clinical information from 260,000 patients suffering from 51 common diseases, with a minimum of 5800 screening information being accessible for research. The study utilized GWAS summary statistics to incorporate SNPs linked to IBD, UC, CD, HCC, CCA, and PC in both European and East Asian ancestry. Therefore, there was no need for ethical approval.

### 2.2 GWAS summary statistics of IBD

GWAS summary data for IBD and its subtypes CD and UC were acquired from the IIBDGC. The IBD cohort included 65,642 Europeans and 6543 East Asians, while the CD cohort included 51,874 Europeans and 5409 East Asians, and the UC cohort included 47,745 Europeans and 4583 East Asians. (Table 1).

**TABLE 1:** Characteristics of the study population.

Traits	Data sources	Case/control	Sample size	Population	Gender	F statistic (total)
Exposure						
Inflammatory bowel disease	IIBDGC	31,665/33977	65,642	European	Males and Females	806.77
Crohn's disease	IIBDGC	17,897/33977	51,874	European	Males and Females	1574.42
Ulcerative colitis	IIBDGC	13,768/33977	47,745	European	Males and Females	1044.86
Inflammatory bowel disease	IIBDGC	2824/3719	6543	East Asian	Males and Females	524.38
Crohn's disease	IIBDGC	1690/3719	5409	East Asian	Males and Females	6808.17
Ulcerative colitis	IIBDGC	1134/3719	4853	East Asian	Males and Females	691.50
Outcome						
Hepatocellular carcinoma	UK Biobank	168/372016	372,184	European	Males and Females	NA
Cholangiocarcinoma	UK Biobank	350/372016	372,366	European	Males and Females	NA
Pancreatic cancer	PanScan1	1896/1939	3835	European	Males and Females	NA
Hepatocellular carcinoma	BioBank Japan	1866/195745	197,611	East Asian	Males and Females	NA
Cholangiocarcinoma	BioBank Japan	339/195745	196,084	East Asian	Males and Females	NA
Pancreatic cancer	BioBank Japan	442/195745	196,187	East Asian	Males and Females	NA

### 2.3 GWAS summary statistics of hepato‐pancreato‐biliary cancers

Given that this study sought to explore the potential association between IBD and HPBC, we examined summary data from UK Biobank, BioBank Japan, and PanScan1 databases. Our analysis of HCC summary statistics involved 372,184 Europeans (UK Biobank) and 197,611 East Asians (BioBank Japan). For CCA summary statistics, we considered 372,366 Europeans (UK Biobank) and 196,084 East Asians (BioBank Japan), while PC summary statistics were based on 3835 Europeans (PanScan1) and 196,187 East Asians (BioBank Japan), as presented in Table 1.

### 2.4 Selection and validation of instrumental variables

**FIGURE 1:**
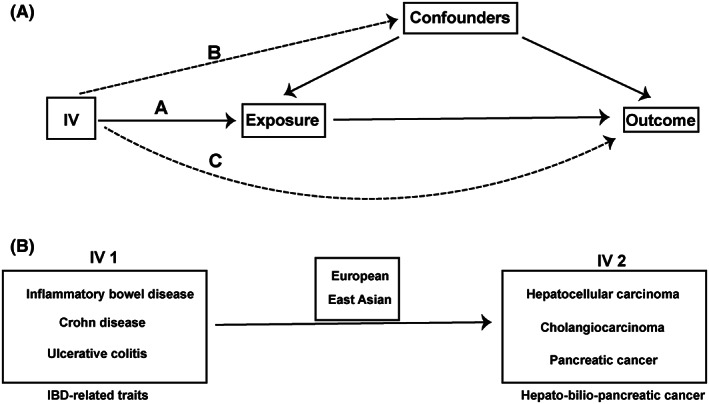
The basic principles of Mendelian randomization (MR) study. (A) represents the three principal assumptions; (B) represents the one‐way MR design. IVs, instrumental variables; IBD, inflammatory bowel disease.

It has been established that the following three criteria must be met for independent genetic variants as IVs in MR studies^18^: (1) IVs are closely related to exposure; (2) There is no pleiotropic association between IVs and any potential confounders; (3) IVs have no direct effect on the outcome except affecting the outcome by associated exposure (Figure 1). The latter two criteria are unrelated to pleiotropy. To satisfy the first criteria, we selected SNPs (r^2^ < 0.1) with a high correlation with the exposure factor and without linkage disequilibrium, reaching statistical significance (*p* < 5 × 10^−8^). Moreover, SNPs with a strong correlation (r^2^ > 0.8) were used as proxies for SNPs for which the effect estimate could not be found in the GWAS summary statistics. According to the PhenoScanner database, these SNPs as IVs were examined for possible violation of the above criteria (2) and (3), excluding SNPs closely related to the occurrence of hepatobiliary pancreatic tumors (BMI, smoking, drinking, diabetes, and viral infection, etc.).

To assess whether the above SNPs were appropriate IVs, we utilized the MR‐Egger method^20^ to evaluate the presence of horizontal pleiotropy in the selected SNPs. If the intercept deviates from the origin, it indicates a potential pleiotropic effect of IVs, characterized by a *p* value <0.05 for the intercept term. A *p* value ≥0.05 for the intercept term indicated the absence of horizontal pleiotropy for the selected instrumental variables.

*F*‐statistics are commonly used to assess the strength of the correlation between instrumental variables and exposure. The equation of *F*‐statistic is $F=\left(\frac{R2}{1-R2}\right)*\left(\frac{n-k-1}{k}\right)$. *R* ^*2*^ represents the exposure variance interpreted by the selected SNPs, *n* is the number of samples, and *k* is the number of IVs included. An *F* value less than 10 indicated a weak correlation between the included IV and exposure, and this IV was removed.^21^

### 2.5 Statistical analysis

A two‐sample one‐way Mendelian Randomization analysis was conducted to explore the potential causal relationship between IBD (including its subtypes) and HPBC. We used the inverse‐variance weighted (IVW) method with multiplicative random effects for estimating causality between exposure and outcome. This method was the most reliable index (P for MR‐Egger intercept >0.05) without direct evidence of gene pleiotropy in the selected IVs. The causal effect of each SNP was estimated by dividing the corresponding outcome effect size by the exposure effect size. Cochran's Q test was used to estimate the degree of heterogeneity among the instrumental variables.^22^ If the heterogeneity was not significant (*p* < 0.05), the fixed effects model was used; otherwise, the inverse‐variance weighted method with the multiplicative random effects model was used.^23^ In addition, we performed pleiotropy testing using the Robust Adjusted Profile Score (RAPS), which is more powerful than the traditional MR approach because of the use of a random‐effects distribution to model the pleiotropic genetic effects.^24^ A *p* value <0.05 was statistically significant. All analyses in the present study were performed using the open‐source statistical software R (version 4.0.2) and the “TwoSampleMR” package (version 0.5.6).

### 2.6 Sensitivity analysis (SA)

We performed multiple sensitivity analyses to validate the Mendelian Randomization causal effect estimates.

First, we tested for potential pleiotropy of IVs using the MR‐Egger method, and pleiotropic correction for causal effects could be obtained by estimating the slope of the MR‐Egger regression. Besides conducting sensitivity analysis, we used the weighted median estimator (WME) to evaluate the accuracy of MR estimates. Then, a Robust Adjusted Profile Score analysis was conducted to strengthen our results since some weaker IVs may have been included. Moreover, we used MR‐PRESSO^25^ to identify and remove possible pleiotropic instrumental variables; adjusted estimates obtained with MR‐PRESSO were used as the main indicator of causal effect estimates if horizontal pleiotropy was present. In addition, the Leave‐one‐out method was applied. In this respect, after excluding each SNP, the effect estimation of the remaining SNPs was examined to determine the influence of nonspecific SNPs on the causal association.^26^ The results of this MR study were deemed sensitive if they demonstrated that no single SNP significantly affected the overall causal estimates obtained for all instrumental variables.

## 3 RESULTS

### 3.1 SNP selection

We found that for IBD and its subtypes CD and UC, the European population exhibited a detection of 130, 115, and 86 SNPs, whereas the East Asian population had a detection of 11, 14, and 9 SNPs, respectively (Table S1–S6).

No weak instrumental variables were found in the exposure factors, and all *F*‐statistics were higher than 10, indicating that the bias caused by “weak” instrumental variables was small. The IBD *F*‐statistics for European and East Asian populations were 806.77 and 524.38, respectively, while those for CD were 1574.42 (European) and 6808.17 (East Asian). For UC, the *F*‐statistics were 1044.86 in the European population and 691.50 in the East Asian population (Table 1).

### 3.2 Causal relationship analysis between IBD and HPBC

#### 3.2.1 Analysis of the European population

It is well‐established that IBD contributes to the risk of PC and that a causal association exists between them. Based on IVW analysis, PC incidence was approximately 1.22‐fold higher than in the general population in IBD populations (OR = 1.2215, 95% CI: 1.0022–1.4888, *p* = 0.0475). Significant results were obtained for Mendelian randomization‐Egger (MR‐Egger) (*p* = 0.2685) and Cochran's Q test (*p* = 0.52242) (>0.05), which indicated no bias and heterogeneity. Moreover, the RAPS suggested that IBD contributed to the risk of PC (OR = 1.2255, 95% CI: 1.0018–1.4991, *p* = 0.0480).

In this study, CD was a risk factor for PC, and the two were causally related. IVW analysis revealed that when CD was an exposure factor, the incidence of PC was about 1.14‐fold higher than the general population. No statistically significant results (*p* > 0.05) were obtained for MR‐Egger regression and Cochran's Q test (*p* = 0.1392 and *p* = 0.6306), implying that there was no bias and heterogeneity. In the European population, weighted median (OR = 1.3695, 95% CI: 1.1154–1.6816, *p* = 0.0027), RAPS (OR = 1.1459, 95% CI: 1.0013–1.3112, *p* = 0.0478), and MR‐PRESSO (OR = 1.1443, 95% CI: 1.0080–1.2990, *p* = 0.0442) indicated that CD contributed to the risk of PC.

In the European population, neither IBD nor CD increased the risk of HCC (OR = 1.0000, 95% CI: 0.9999–1.0001, *p* = 0.8127), (OR = 1.0000, 95% CI: 0.9999–1.0001, *p* = 0.7491) and CCA (OR = 1.0000, 95% CI: 0.9999–1.0001, *p* = 0.8807), (OR = 1.0000, 95% CI: 0.9999–1.0001, *p* = 0.8058). UC did not increase the risk of HCC (OR = 1.0000, 95% CI: 0.9999–1.0001, *p* = 0.6444), CCA (OR = 1.0000, 95% CI: 0.9999–1.0001, *p* = 0.8540), and PC (OR = 1.0240, 95% CI: 0.8457–1.2401, *p* = 0.8075).

**FIGURE 2:**
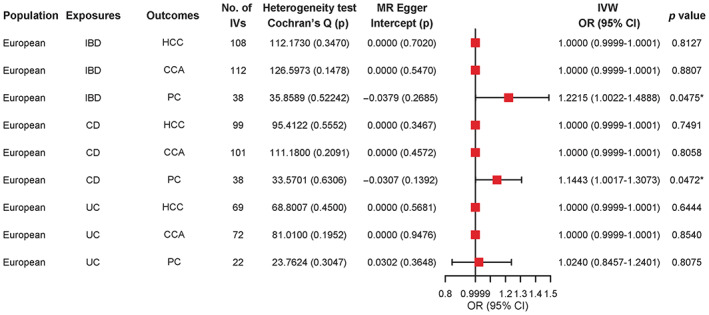
Mendelian randomization (MR) results for European population, with HCC, CCA, and PC as outcomes. IBD, inflammatory bowel disease; CD, crohn's disease; UC, ulcerative colitis; HCC, hepatocellular carcinoma; CCA, cholangiocarcinoma; PC, pancreatic cancer; IVs, instrumental variables; IVW, inverse‐variance weighted; OR, odds ratio; 95% CI, 95% confidence interval.

The above results are shown in detail in Figure 2 and Table 2.

**TABLE 2:** MR results of weighted‐median, RAPS, and MR‐PRESSO methods for pleiotropy testing in European population.

Population	Exposures	Outcomes	No. of IVs	Weighted median		RAPS		MR‐PRESSO	
				OR (95% CI)	*p* value	OR (95% CI)	*p* value	OR (95% CI)	*p* value
European	IBD	HCC	108	1.0000 (0.9998, 1.0001)	0.8645	1.0000 (0.9999,1.0001)	0.8126	1.0000 (0.9999, 1.0001)	0.8175
European	IBD	CCA	112	1.0000 (0.9997, 1.0002)	0.8141	1.0000 (0.9999,1.0001)	0.8805	1.0000 (0.9999, 1.0002)	0.8885
European	IBD	PC	38	1.0898 (0.8190, 1.4500)	0.5552	1.2255 (1.0018,1.4991)	0.0480*	1.2215 (1.0053, 1.4842)	0.0514
European	CD	HCC	99	1.0000 (0.9999, 1.0002)	0.6073	1.0000 (0.9999,1.0001)	0.6003	1.0000 (0.9999, 1.0001)	0.7465
European	CD	CCA	101	1.0000 (0.9999, 1.0002)	0.6252	1.0000 (0.9999,1.0001)	0.7218	1.0000 (0.9999, 1.0001)	0.8161
European	CD	PC	38	1.3695 (1.1154, 1.6816)	0.0027*	1.1459 (1.0013,1.3112)	0.0478*	1.1443 (1.0080, 1.2990)	0.0442*
European	UC	HCC	69	1.0000 (0.9998, 1.0001)	0.6390	1.0000 (0.9999,1.0001)	0.5992	1.0000 (0.9999, 1.0001)	0.6478
European	UC	CCA	72	1.0000 (0.9997, 1.0002)	0.7488	1.0000 (0.9999,1.0002)	0.7692	1.0000 (0.9998, 1.0001)	0.8637
European	UC	PC	22	0.9665 (0.7308, 1.2781)	0.8109	1.0245 (0.8437,1.2439)	0.8072	1.0241(0.8354, 1.2553)	0.8211

Abbreviations: 95% CI, 95% confidence interval; CCA, cholangiocarcinoma; CD, crohn's disease; HCC, hepatocellular carcinoma; IBD, inflammatory bowel disease; IVs, instrumental variables; MR, mendelian randomization; MR‐PRESSO, Mendelian Randomization Pleiotropy Residual Sum and Outlier; OR, odds ratio; PC, pancreatic cancer; RAPS, Robust Adjusted Profile Score; UC, ulcerative colitis.

Bold values and * indicate a *p* value < 0.05.

#### 3.2.2 Analysis of the East Asian population

IBD is a risk factor for HCC, and a causal relationship was found between them. IVW analysis showed that the incidence of HCC in IBD (OR = 1.2777, 95% CI: 1.0709–1.5244, *p* = 0.0065) as an exposure factor was 1.28‐fold higher than in the normal population. No significant results were obtained for MR‐Egger regression (*p* value 0.9487), suggesting the absence of bias, although significant heterogeneity was observed (Cochran's Q test *p* value <0.0065). The RAPS (OR = 1.3021, 95% CI: 1.1953–1.4184, *p* < 0.0001) and MR‐PRESSO (OR = 1.1350, 95% CI: 0.9524–1.3527, *p* = 0.0236) further revealed that IBD contributed to the risk of HCC.

The development of HCC and CCA has been attributed to UC as a risk factor, establishing a definite causal relationship. IVW analysis showed that when UC was an exposure factor, the incidence rate of HCC (OR = 1.12365, 95% CI: 1.1466–1.3334, *p* < 0.0001) and CCA (OR = 1.3107, 95% CI: 1.0983–1.5641, *p* = 0.0027) were 1.12 and 1.31 times higher than that of the normal population, respectively. The *p* value of MR‐Egger regression was 0.2927, and the *p* value of Cochran's Q test was 0.1392, suggesting an absence of bias and heterogeneity. RAPS, MR‐PRESSO, and weighted median indicated that UC was an independent risk factor for HCC (OR = 1.2395, 95% CI: 1.1515–1.3341, *p* < 0.0001), (OR = 1.2365, 95% CI: 1.1235–1.3608, *p* = 0.0049), (OR = 1.2388, 95% CI: 1.1087–1.3841, *p* = 0.0002) and CCA (OR = 1.3491, 95% CI: 1.1393–1.5977, *p* = 0.0005), (OR = 1.3107, 95% CI: 1.1551–1.4872, *p* = 0.0057), (OR = 1.3118, 95% CI: 1.0548–1.6314, *p* = 0.0147).

We established that CD was a risk factor for PC, and a causal relationship was detected. IVW analysis showed that the PC incidence rate was 1.19‐fold higher in CD populations than in the normal population (OR = 1.1876, 95% CI: 1.0741–1.3132, *p* = 0.0008). No significant results were obtained for the MR‐Egger regression (*p* = 0.1820) and Cochran's Q test (*p* = 0.7811), indicating no bias and heterogeneity. RAPS (OR = 1.1891, 95% CI: 1.0725–1.3184, *p* = 0.0010), MR‐PRESSO (OR = 1.1876, 95% CI: 1.0929–1.2906, *p* = 0.0014), and weighted median (OR = 1.1784, 95% CI: 1.0261–1.3533, *p* = 0.0201) were tested for pleiotropy, and it was confirmed that CD was a risk factor for PC.

Besides, IBD did not increase the risk of CCA (OR = 1.1431, 95% CI: 0.9413–1.3881, *p* = 0.1772) and PC (OR = 1.1479, 95% CI: 0.9687–1.3602, *p* = 0.1112) in East Asians. Consistently, CD did not increase the risk of HCC (OR = 1.0394, 95% CI: 0.9509–1.1361, *p* = 0.3946) and CCA (OR = 0.9390, 95% CI: 0.8369–1.0534, *p* = 0.2833). Additionally, UC did not increase the risk of PC (OR = 0.9836, 95% CI: 0.8429–1.1478, *p* = 0.8339).

**FIGURE 3:**
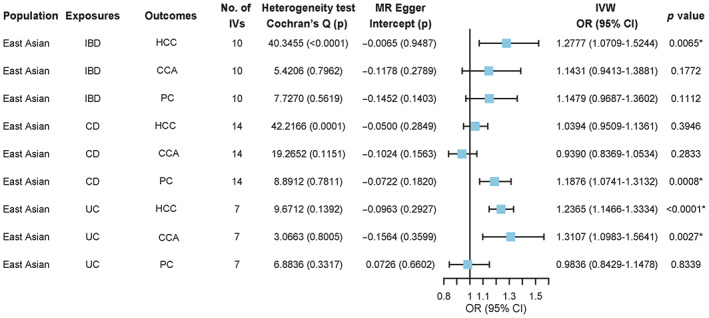
Mendelian randomization (MR) results for East Asian population, with HCC, CCA, and PC as outcomes. IBD, inflammatory bowel disease; CD, crohn's disease; UC, ulcerative colitis; HCC, hepatocellular carcinoma; CCA, cholangiocarcinoma; PC, pancreatic cancer; IVs, instrumental variables; IVW, inverse‐variance weighted; OR, odds ratio; 95% CI, 95% confidence interval.

The above results are detailed in Figure 3 and Table 3.

**TABLE 3:** MR results of weighted‐median, RAPS, and MR‐PRESSO methods for pleiotropy testing in East Asian population.

Population	Exposures	Outcomes	No. of Ivs	Weighted median		RAPS		MR‐PRESSO	
				OR (95% CI)	*p* value	OR (95% CI)	*p* value	OR (95% CI)	*p* value
East Asian	IBD	HCC	10	1.0942 (0.9388, 1.2752)	0.2493	1.3021 (1.1953, 1.4184)	<0.0001*	1.1350 (0.9524, 1.3527)	0.0236*
East Asian	IBD	CCA	10	1.2631 (0.9706, 1.6437)	0.0822	1.1444 (0.9380, 1.3961)	0.1837	1.1431 (0.9831, 1.329)	0.1161
East Asian	IBD	PC	10	1.1964 (0.9635, 1.4856)	0.1046	1.1494 (0.9659, 1.3676)	0.1166	1.1479 (0.9808, 1.3433)	0.1197
East Asian	CD	HCC	14	1.0568 (0.9812, 1.1383)	0.1448	1.0420 (0.9923, 1.0943)	0.0991	1.0394 (0.9509, 1.1361)	0.4100
East Asian	CD	CCA	14	1.0120 (0.8606, 1.1899)	0.8855	0.9378 (0.8346, 1.0537)	0.2799	0.9390 (0.8163, 1.0801)	0.3941
East Asian	CD	PC	14	1.1784 (1.0261, 1.3533)	0.0201*	1.1891 (1.0725, 1.3184)	0.0010*	1.1876 (1.0929, 1.2906)	0.0014*
East Asian	UC	HCC	7	1.2388 (1.1087, 1.3841)	0.0002*	1.2395 (1.1515, 1.3341)	<0.0001*	1.2365 (1.1235, 1.3608)	0.0049*
East Asian	UC	CCA	7	1.3118 (1.0548, 1.6314)	0.0147*	1.3491 (1.1393, 1.5977)	0.0005*	1.3107 (1.1551, 1.4872)	0.0057*
East Asian	UC	PC	7	0.9238 (0.7658, 1.1143)	0.4073	0.9935 (0.8588, 1.1492)	0.9296	0.9836 (0.8337, 1.1605)	0.8513

Abbreviations: 95% CI, 95% confidence interval; CCA, cholangiocarcinoma; CD, crohn's disease; HCC, hepatocellular carcinoma; IBD, inflammatory bowel disease; IVs, instrumental variables; MR, mendelian randomization; MR‐PRESSO, Mendelian Randomization Pleiotropy Residual Sum and Outlier; OR, odds ratio; PC, pancreatic cancer; RAPS, Robust Adjusted Profile Score; UC, ulcerative colitis. 1

### 3.3 Sensitivity analysis

Figure S1 and S2 demonstrates that the results of this MR Study were sensitive, as sensitivity analysis using the Leave‐one‐out technique showed that none of the SNPs had a significant impact on the causal estimates of all instrumental variables.

## 4 DISCUSSION

It is well‐established that IBD subjects are at increased risk of bowel cancer.^27^ Interestingly, an increasing body of evidence from recently published studies suggests that the risk of extraintestinal cancer is significantly increased in this patient population. Although ample literature substantiates that IBD and its subtypes CD and UC increase the risk of HCC, CCA, and PC,^16^, ^17^ this evidence comes from observational and in vivo studies, and the conclusions may be affected by various confounding factors. Accordingly, the causal association between IBD and HPBC remains largely unclear, warranting further research. Unlike prior studies, the present study established a causal link between IBD and HPBC and discovered differences in genetic susceptibility across European and East Asian populations. Mendelian Randomization studies are less likely to be influenced by confounding and exposure factors than observational and in vivo studies.^28^

During analysis of the causal association between IBD (including its subtype CD) and PC, we found that in Europe, when IBD and its subtype CD were used as exposure factors, the risk of PC was 1.22‐ and 1.14‐fold higher than in the normal population, respectively. The PC incidence in the East Asian population with CD as the exposure factor was 1.19‐fold higher than in the normal population. Consistently, an observational study by Åsa H Everhov et al.^29^ substantiated that the overall risk of PC was significantly higher in IBD patients (HR = 1.43, 95% CI: 1.30–1.58). In this regard, an increased risk of PC was observed in CD (HR = 1.44, 95% CI: 1.18–1.74) and UC (HR = 1.35, 95% CI: 1.19–1.53) patients compared with the general population. Two recent studies have found that IL‐6 and IL‐18 play a key role in the pathogenesis of IBD and PC via a common pathogenic pathway. Li et al.^30^ corroborated that interleukin‐18 (IL‐18) could play an important role in both CD and PC, given its involvement in T helper type 1 (Th1) and Th2 immune responses and the activation of NK cells and macrophages. Using sgp130Fc protein or sgp130Fc transgenic mouse model, Jürgen Scheller et al.^31^ demonstrated that cross signaling of interleukin‐6 (IL‐6) through soluble IL‐6R is a key factor in the pathogenesis of IBD and PC.

We discovered a causal link between IBD, specifically its subtype UC, and HCC. Our study found that in the East Asian population, using IBD as an exposure factor resulted in a 1.28‐fold increase in the incidence of HCC compared with the general population. When UC was used as an exposure factor, the incidence of HCC was 1.12 times higher than that of the normal population. There is a rich literature available substantiating that HCC risk in IBD patients is higher than in the general population.^32^, ^33^, ^34^ Interestingly, another study^35^ found that IBD and HCC share common immune‐related biomarkers. They performed differential gene expression analyses and found that CXCL2, MMP9, SPP1, and SRC are key genes in IBD and HCC. In addition, several transcription factors (FOXC1, FOXL1, GATA2, YY1, ZNF354C, and TP53) and miRNA (miR‐124‐3p, miR‐1‐3p, miR‐7‐5p, miR‐34a‐5p, and miR‐99b‐5p) were identified that might mediate the expression of these key genes. It is now understood that IBD patients with inflamed colons exhibit a high expression of CXCL2, which activates ERK1/2 and controls the proliferation of HCC cells.^36^, ^37^, ^38^ The MMP9 gene is upregulated in inflamed mucosa or serum of patients with IBD and is a novel marker of inflammation in the intestine.^39^ In HCC patients, MMP9 is associated with tumor invasion and adverse outcomes.^40^ In addition, IBD has been associated with the upregulation of the SPP1 gene.^41^ Polymorphisms in the SPP1 gene have also been linked to HCC.^42^, ^43^ Besides, SRC is involved in the progression, invasion, and metastasis of HCC.^44^, ^45^ Overall, the above findings further support the link between IBD and HCC.

During analysis of the causal relationship between UC and CCA, we found that when UC was used as an exposure factor in the East Asian population, the incidence of CCA was 1.31‐fold higher than in the normal population. Primary sclerosing cholangitis (PSC) is a chronic cholestatic liver disease. Bile acids produced by cholestasis lead to decreased PH, increased apoptosis, and activation of the ERK1/2, Akt, and NF‐κB pathways, promoting cell proliferation, migration, and survival. Studies have shown that IBD (including CD and UC) is closely related to PSC and can lead to bile duct cells being exposed to inflammatory cytokines (including IL‐6, TNF‐α, Cox‐2, and Wnt, etc.),^46^ causing cholestasis and progressive mutations in tumor suppressor genes, proto‐oncogenes, and DNA mismatch repair genes. IBD is one of the key risk factors of bile duct cancer.^47^, ^48^, ^49^, ^50^ In addition, immunosuppression due to IBD treatment may also a factor in IBD‐associated carcinogenesis.^51^

In addition, some scholars have conducted research from the perspective of intestinal flora microecology and have made some crucial discoveries. Several studies have identified gene variants (including NOD2, ATG16L1, CARD9, and CLEC7A) that affect gut microbial immune response in IBD patients and uncovered that these gene variants could induce intestinal microecological dysbiosis.^52^, ^53^, ^54^ Notably, our literature review revealed that gut dysbiosis might lead to the development and progression of PC, HCC, and CCA.^55^, ^56^, ^57^, ^58^, ^59^, ^60^, ^61^

As a result, it is reasonable to conclude that IBD is a chronic inflammatory disease not limited to the bowel, and its subtypes can increase the risk of HPBC, exhibiting a causal association.

IBD and HPBC are diseases with complex pathogenesis and significant genetic risk differences between populations. Our study found a causal association between IBD and HPBC in the East Asian population but only between IBD and PC in Europeans. The reason for this causal relationship among different populations remains unclear. Genetic diversity may be the key to unraveling the genetic relationship between different populations. Although the past decade has witnessed unprecedented progress in identifying genetic variants that affect human diseases, most genetic risks remain unexplained, warranting further studies to find novel biological evidence on how IBD affects the risk of HPBC.

Importantly, our findings provide the theoretical basis for precancer screening and intervention. However, there are some limitations to our study. First, the key assumptions of MR have limitations since it is difficult to guarantee that any confounding factors or any potential pleiotropic effects do not influence the relationship between exposure and outcome. Second, we used GWAS summary data, which may have been affected by heterogeneity in quality control and selection criteria. Third, the principle of MR research is that causality can be inferred from the genetic level; however, we can only determine the underlying causality, not the specific biological pathways that induce it. Fourth, our findings were obtained from an East Asian and European cohort, so they are not generalizable to other ethnic groups. Fifth, the database does not divide IBD (including CD and UC) or hepatobiliary and pancreatic tumors into male and female patients, and we do not have access to the original data for further analysis. In the future, we hope to have more access to the original GWAS data or wait for the database update to conduct additional analysis and explore the effect of IBD on the incidence of hepatobiliary and pancreatic tumors by gender. Finally, we could not determine whether HPBC could induce an increase in IBD incidence using a two‐way MR study due to the lack of suitable SNPs. Consequently, reverse causality may affect our conclusions. Accordingly, more SNP data are required in the future to increase the robustness of our findings.

## 5 CONCLUSIONS

The present study found that the European population showed a causal relationship with PC when IBD and its subtype CD were used as exposure factors, with an increase in PC incidence. Meanwhile, in the East Asian population, the risk of HCC increased when IBD was used as an exposure factor. Moreover, the incidence of HCC and CCA increased when UC was used as an exposure factor, while the incidence of PC increased when CD was used as an exposure factor. As a result, IBD patients and their physicians emphasize HPBC screening and prevention. Collectively, our findings are clinically relevant and might contribute to improved prevention, interdisciplinary research, and overall patient care. Further research is nevertheless needed to determine the pathophysiological pathways related to HPBC in IBD patients.

## AUTHOR CONTRIBUTIONS

**Jinsheng Huang:** Conceptualization (lead); data curation (lead); formal analysis (lead); investigation (lead); methodology (lead); project administration (lead); resources (lead); validation (lead); visualization (lead); writing – original draft (lead). **Xujia Li:** Formal analysis (equal); software (equal). **Jicheng Hong:** Investigation (equal); supervision (equal). **Lingli Huang:** Data curation (supporting); software (supporting). **Qi Jiang:** Data curation (supporting); formal analysis (supporting); software (supporting). **Shunqi Guo:** Formal analysis (supporting); supervision (supporting). **Yuming Rong:** Data curation (equal); project administration (equal); writing – review and editing (equal). **guifang Guo:** Conceptualization (lead); funding acquisition (lead); writing – review and editing (lead).

## FUNDING INFORMATION

This study was funded by the Guangdong Provincial Natural Science Foundation (grant no. 2021A1515012368).

## CONFLICT OF INTEREST STATEMENT

No financial conflict or other relationships for each author to be declared.

## Supporting information


Figure S1.

Figure S2.

Table S1–S6.

## Data Availability

All data generated or analyzed during this study are included within the article.

## Footnotes

1 Bold values and * indicate a *p* value < 0.05.
