# Supplementary figures and images for: Inflammatory bowel disease increases the risk of hepatobiliary pancreatic cancer: A two‐sample Mendelian randomization analysis of European and East Asian populations
Source: Cancer Med. 2023 May 15;12(12):13599–609. doi: 10.1002/cam4.6057 (PMC10315785; doi:10.1002/cam4.6057)

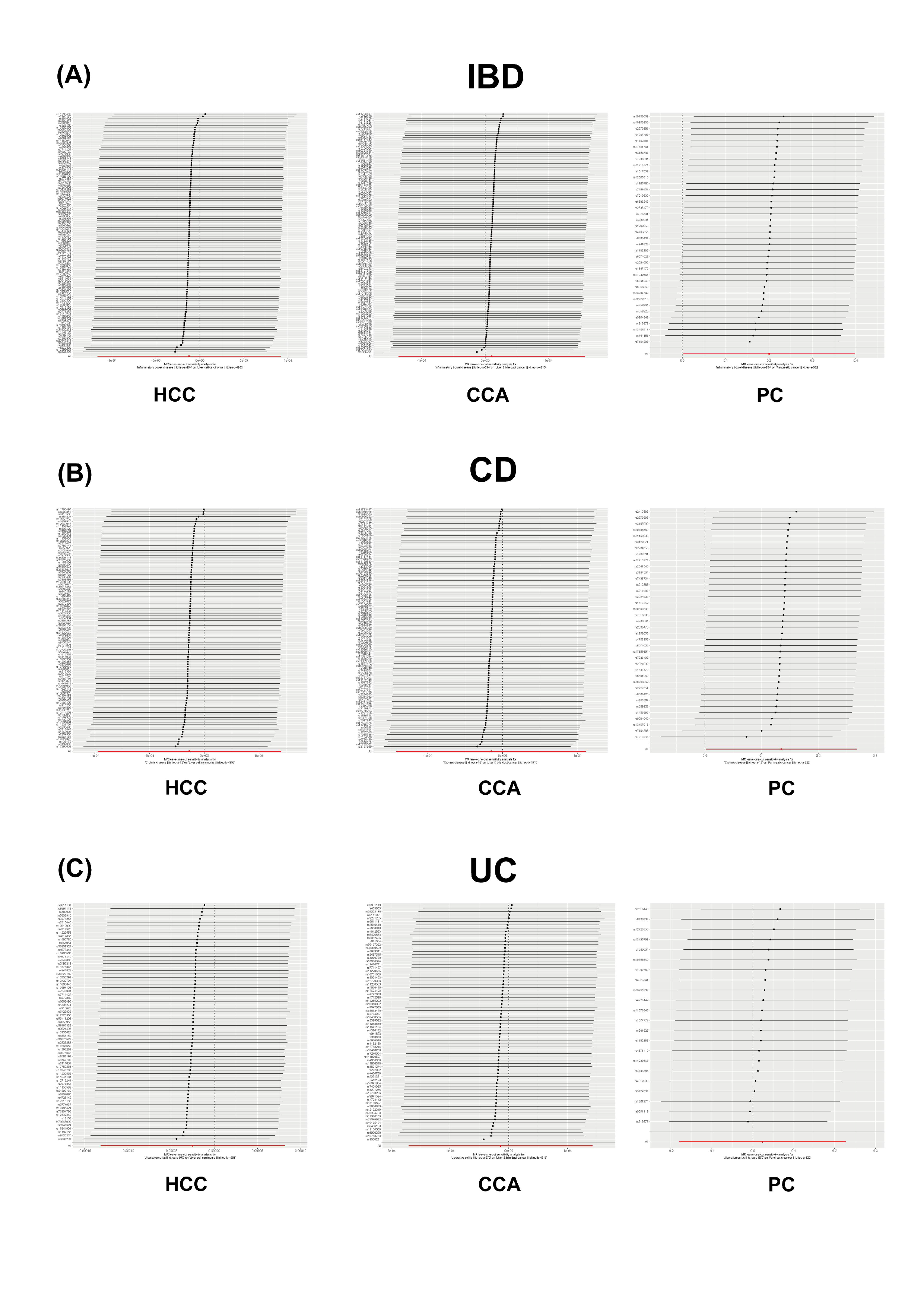

Supplement: Supplementary file 1 — Figure S1. [file CAM4-12-13599-s003.tif]

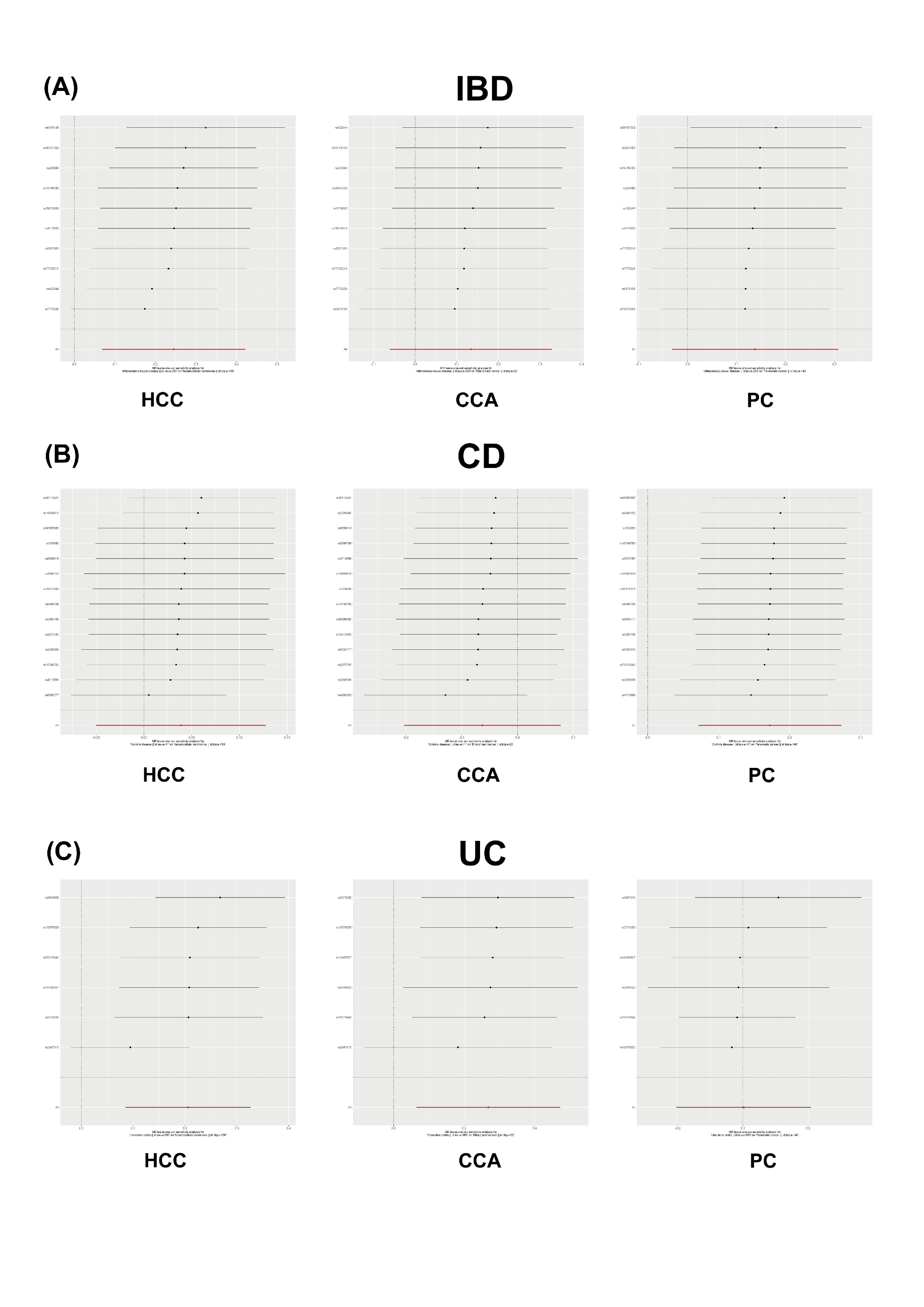

Supplement: Supplementary file 2 — Figure S2. [file CAM4-12-13599-s002.tif]
